# Gender-Divergent Profile of Bile Acid Homeostasis during Aging of Mice

**DOI:** 10.1371/journal.pone.0032551

**Published:** 2012-03-05

**Authors:** Zidong Donna Fu, Iván L. Csanaky, Curtis D. Klaassen

**Affiliations:** Department of Pharmacology, Toxicology, and Therapeutics, University of Kansas Medical Center, Kansas City, Kansas, United States of America; Governmental Technical Research Centre of Finland, Finland

## Abstract

Aging is a physiological process with a progressive decline of adaptation and functional capacity of the body. Bile acids (BAs) have been recognized as signaling molecules regulating the homeostasis of glucose, lipid, and energy. The current study characterizes the age-related changes of individual BA concentrations by ultra-performance liquid chromatography-tandem mass spectrometry (UPLC-MS/MS) in serum and liver of male and female C57BL/6 mice from 3 to 27 months of age. Total BA concentrations in serum increased 340% from 3 to 27 months in female mice, whereas they remained relatively constant with age in male mice. During aging, male and female mice shared the following changes: (1) BA concentrations in liver remained relatively constant; (2) the proportions of beta-muricholic acid (βMCA) increased and deoxycholic acid (DCA) decreased between 3 and 27 months in serum and liver; and (3) total BAs in serum and liver became more hydrophilic between 3 and 27 months. In female mice, (1) the mRNAs of hepatic BA uptake transporters, the Na^+^/taurocholate cotransporting polypeptide (Ntcp) and the organic anion transporting polypeptide 1b2 (Oatp1b2), decreased after 12 months, and similar trends were observed for their proteins; (2) the mRNA of the rate-limiting enzyme for BA synthesis, cholesterol 7α-hydroxylase (Cyp7a1), increased from 3 to 9 months and remained high thereafter. However, in male mice, Ntcp, Oatp1b2, and Cyp7a1 mRNAs remained relatively constant with age. In summary, the current study shows gender-divergent profiles of BA concentrations and composition in serum and liver of mice during aging, which is likely due to the gender-divergent expression of BA transporters Ntcp and Oatp1b2 as well as the synthetic enzyme Cyp7a1.

## Introduction

Aging has become one of the most important global issues, because the elderly population (with chronological age of 65 years and older) are increasing, and it is estimated they will reach 22% of the population in 2050. Elderly people have an increased incidence of various age-related diseases, including liver and gastrointestinal (GI) diseases. The prevalence of chronic liver disease increases in the elderly, such as alcoholic liver disease, non-alcoholic fatty liver disease, viral hepatitis C, as well as hepatocellular carcinoma [1]. In addition, the risk of stomach cancer increases with age, and more than 90% of colon cancers were found in people over 50-years of age.

**Figure 1:**
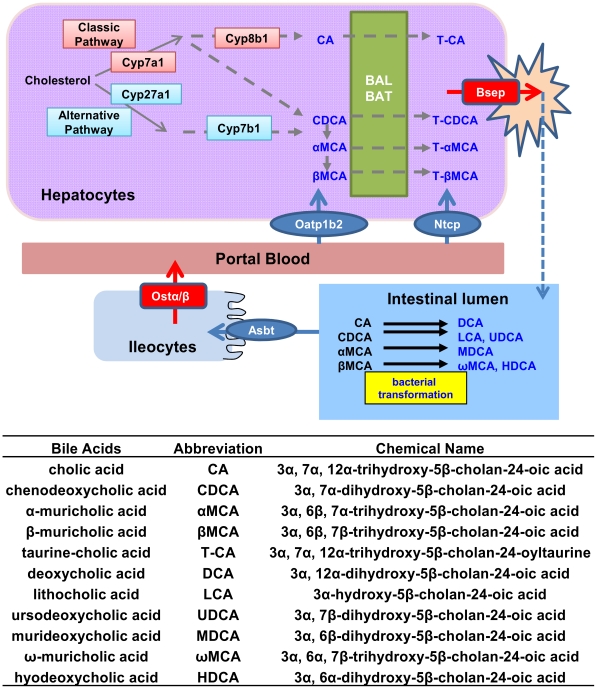
Scheme of the enterohepatic circulation (EHC) of bile acids (BAs) in mice. Primary BAs are synthesized and conjugated in hepatocytes, and secreted into intestine to facilitate the fat digestion. Cyp7a1 is the rate-limiting enzyme for BA biosynthesis. Primary BAs are conjugated mainly with taurine in mice, by conjugating enzymes BAL and BAT. BAs are excreted into the canalicular bile by Bsep. In the intestinal lumen, BAs are transformed into secondary BAs by intestinal bacteria. BAs are efficiently reabsorbed by Asbt into ileocytes and effluxed into the portal blood by Ostα/β. Ntcp and Oatp1b2 take up BAs into hepatocytes. CA, CDCA, αMCA, and βMCA are the primary BAs in mice. DCA is the secondary BA of CA, LCA and UDCA are secondary BAs of CDCA, and ωMCA, MDCA, and HDCA are secondary BAs of α,βMCA.

In the enterohepatic system, bile acids (BAs) play multifaceted physiological functions. Apart from their well-known roles for dietary lipid absorption and cholesterol homeostasis, BAs are increasingly appreciated as complex metabolic signaling molecules [2], regulating glucose, lipid, and energy metabolism. In humans, up to 95% of the BAs are efficiently recycled daily through the “enterohepatic circulation” (EHC) (Fig. 1), and only 5% are newly synthesized. Primary BAs are synthesized in the liver, namely cholic acid (CA) and chenodeoxycholic acid (CDCA) in humans. In rodents, CDCA can be hydroxylated into alpha-muricholic acid (αMCA), which is converted to beta-muricholic acid (βMCA) by 7-OH epimerization. In intestine, bacterial transformation of primary BAs occur to synthesize secondary BAs. CA is converted to its secondary BA deoxycholic acid (DCA), CDCA to lithocholic acid (LCA) and ursodeoxycholic acid (UDCA), αMCA to murideoxycholic acid (MDCA), and βMCA to ω-muricholic acid (ωMCA) and hyodeoxycholic acid (HDCA) [3], [4].

Cholesterol 7α-hydroxylase (Cyp7a1) is the rate-limiting enzyme for BA biosynthesis in the liver [5]. Cyp8b1 catalyzes CA synthesis, and thus controls the ratio of CA to CDCA [6]. The alternative synthetic pathway of BA synthesis starts with side-chain oxidation by Cyp27a1 [7] and involves Cyp7b1 [8] to produce CDCA. BAs are conjugated with taurine or glycine by bile acid-CoA ligase (BAL) and bile acid-CoA∶amino acid *N*-acyltransferase (BAT) in liver. Mouse BAT is a taurine-specific conjugating enzyme [9]. BA transporters play important roles in promoting the EHC. In liver, conjugated BAs are taken up from the portal blood by the Na^+^/taurocholate cotransporting polypeptide (Ntcp) [10] and unconjugated BAs by basolateral transporters, such as the organic anion transporting polypeptide 1b2 (Oatp1b2) [11], and BAs and their conjugates are excreted into bile by the bile salt export pump (Bsep) [12]. In ileum, BAs are efficiently reabsorbed by the apical sodium-dependent bile acid transporter (Asbt) [13] and transported to the portal blood by the basolateral heterodimeric organic solute transporter alpha and beta (Ostα/β) [14].

BAs can regulate their own homeostasis [15], [16]. BAs activate their nuclear receptor farnesoid X receptor (FXR) in liver, which transactivates small heterodimer partner (SHP). SHP subsequently forms inactive heterodimer with liver related homologue-1 (LRH-1) [17], resulting in decreased transcription of Cyp7a1. In addition, BAs also activate FXR in the intestine, which induces fibroblast growth factor 15 (Fgf15), an intestinal hormone that travels through the circulation to the liver and down-regulates Cyp7a1 transcription [18], [19].

There is little known about BA homeostasis during aging. Limited reports about BA metabolism in aged humans or rats show inconsistent results. BAs are important signaling molecules, whose homeostasis if disrupted can lead to various age-related diseases, such as metabolic syndrome, cholestatic liver diseases, intestinal bacterial overgrowth and infection, and colorectal cancer. However, BAs are cytotoxic when present in abnormally high concentrations [15]. Therefore, altering BA concentration and composition might be a potential anti-aging intervention.

The current study aims to describe the effect of aging on BA composition and concentration, and determine the molecular mechanism with respect to the expression of genes involved in BA homeostasis in mice. To address these questions, both male and female mice at nine ages, from 3- to 27-months old, were used for serum and tissue collection for BA, mRNA, and protein quantification. Substantial information on the changes of BA concentrations and composition in male and female mice during aging was obtained, as well as possible regulatory mechanisms for these changes.

## Materials and Methods

### Ethics statement

Mice were housed according to guidelines of the Institutional Animal Care and Use Committee at the University of Kansas Medical Center, and procedures were carried out in compliance with standards for use of laboratory animals. Animal experiments performed in this study have been approved by the Institutional Animal Care and Use Committee at the University of Kansas Medical Center (protocol 2011-1969).

### Chemicals and reagents

The sources of individual BA standards and internal standards are described previously by Zhang and Klaassen [16]. Rabbit anti-rat Ntcp antibody (K4), which has cross-reactivity with mouse Ntcp, was a generous gift from Bruno Steiger (University Hospital, Zurich, Switzerland). A polyclonal antibody to mouse Oatp1b2 was developed in our laboratory. β-Actin antibody (ab8227) was purchased from Abcam, Inc. (Cambridge, MA). Goat anti-rabbit IgG horseradish peroxidase-linked secondary antibody was purchased from Sigma-Aldrich (St. Louis, MO). All other chemicals and reagents, unless indicated, were purchased from Sigma-Aldrich (St. Louis, MO).

### Animals

Male and female C57BL/6 mice of various ages were purchased from the National Institute of Aging (Bethesda, MD) and acclimated for at least one month before tissue collections. Mice were housed in an Association for Assessment and Accreditation of Laboratory Animal Care International (AAALAC)-accredited facility with a 14-h light/10-h dark-cycle, temperature-, and humidity-controlled environment and given *ad libitum* access to water and standard rodent chow (Harlan Teklad 8604; Harlan Teklad, Madison, WI). At 3, 6, 9, 12, 15, 18, 21, 24, and 27 months of age, mice (n = 5–7) were anesthetized with pentobarbital (50 mg/kg), and blood was collected from the suborbital vein. After cervical dislocation, liver and ileum (posterior one third of small intestine) were removed, snap-frozen in liquid nitrogen, and stored at −80°C. Tissue collections were between 9:00 and 12:00 in the morning, to decrease the variations due to circadian rhythm of BAs [20]. These studies were approved by the Institutional Animal Care and Use Committee at the University of Kansas Medical Center.

### BA extraction from serum and liver

Internal standards (40 µg/ml d_4_-G-CDCA and 20 µg/ml d_4_-CDCA in MeOH) were added to the samples, and BAs were extracted from liver tissue using methods reported by Zhang and Klaassen [16]. For serum samples, methanol (MeOH) was added for protein precipitation. One ml of MeOH was added to 50 µl of serum spiked with 5 µl IS, vortexed, and centrifuged at 12,000 g for 10 min. The supernatant was aspirated, evaporated under vacuum, and reconstituted in 50 µl of 50% MeOH.

### Quantification of BAs by ultra-performance liquid chromatography-tandem mass spectrometry (UPLC-MS/MS)

The conditions of liquid chromatography and mass spectrometry analysis were previously reported by Zhang and Klaassen [16]. Major individual BAs quantified were T-CA, T-CDCA, T-αMCA, T-βMCA, T-DCA, T-LCA, T-UDCA, T-MDCA, T-ωMCA, T-HDCA, CA, CDCA, αMCA, βMCA, DCA, LCA, UDCA, MDCA, ωMCA, and HDCA. The concentrations of individual BAs were summed to derive the concentrations of conjugated, unconjugated, and total BAs.

### Total RNA Isolation

Total RNA was isolated from liver and ileum tissues using RNA Bee reagent (Tel-Test Inc., Friendswood, TX) following the manufacturer's protocol. The concentration of total RNA in each sample was quantified spectrophotometrically at 260 nm.

### Multiplex Suspension Assay

The mRNA expression of genes of interest in liver was determined by Panomics 2.0 QuantiGene Plex technology (Panomics/Affymetrix Inc., Fremont, CA). Individual gene information can be found on Panomics Web site (http://www.panomics.com) with Panel numbers 21095, 21197, and 21151. Fluorescence was analyzed using a Bio-Plex 200 system array reader with Luminex 100 X-MAP technology, and data were acquired using Bio-Plex data manager software 5.0 (Bio-Rad, Hercules, CA). The mRNA of target genes were normalized to Gapdh.

### Preparation of Crude Membrane Fractions

Livers were homogenized in ST buffer (0.25 M sucrose, 10 mM Tris•HCl, *p*H 7.4) containing protease inhibitors and centrifuged at 100,000 g for 60 min at 4°C. The membrane pellet was rinsed and resuspended with ST buffer with protease inhibitor. Protein concentrations were determined using Pierce protein assay reagents accordingly to the manufacturer's instructions (Pierce Biotechnology, Rockford, IL).

### Western Blot Analysis

Western blots of Ntcp and Oatp1b2 were performed as previously described with minor modifications [21]. Primary antibodies were diluted in blocking buffer as follows: Ntcp (K4, 1∶2000) and Oatp1b2 (1∶1000). Membranes were stripped and reprobed with β-actin antibody (ab8227, 1∶10000) as the loading control. Intensities of protein bands were determined using the Image J software (National Institute of Health).

### Statistical Analysis

Data are presented as mean ± SEM. Data were analyzed by one-way ANOVA, followed by Duncan's post-hoc test, differences being considered significant at *p*<0.05 (daggers † represent differences from 3 months of age in male mice, double daggers ‡ represent differences from 3 months of age in female mice). Asterisks (*) represent gender differences between male and female mice, determined by student t-test (*p*<0.05). Pound signs (#) indicate differences of BA proportions between 3 and 27 months.

## Results

### Total BA Concentrations in Serum during Aging

**Figure 2:**
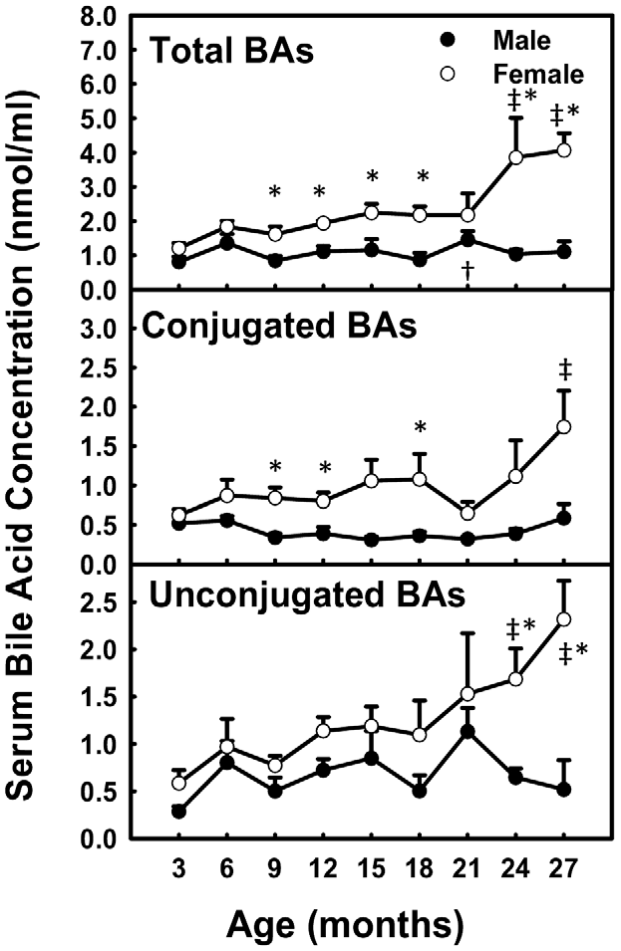
Total BA concentrations in serum during aging of male and female mice. Data are presented as means ± SEM of 5–7 mice. Daggers (†) represent statistically significant difference from the value at 3 months of age during aging of male mice. Double daggers (‡) represent statistically significant difference from the value at 3 months of age during aging of female mice. Age-dependent differences were considered at *p*<0.05 by one-way ANOVA, followed by Duncan's post-hoc test. Asterisks (*) represent statistically significant difference between male and female mice at respective ages during aging (*p*<0.05), by student t-test.

Total BA concentrations in serum remained relatively constant in male mice, whereas in female mice, they increased 340% from 3 to 27 months of age, due to an increase in both conjugated (280%) and unconjugated (400%) BAs (Fig. 2). Female mice had higher total BA concentrations than male mice from 9 (190%) to 27 (370%) months.

### Conjugated BAs in Serum during Aging

**Figure 3:**
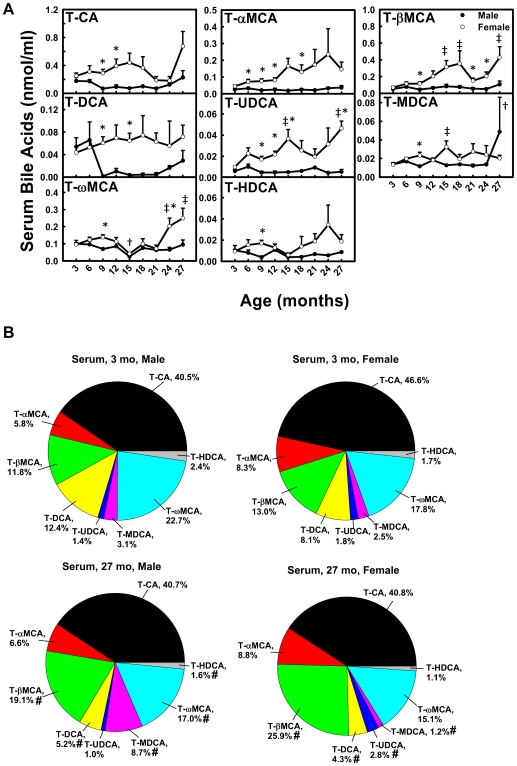
Concentrations of conjugated BAs in serum during aging of male and female mice. (A) Concentrations of individual conjugated BAs in serum during aging and (B) proportions of them in all conjugated BAs in serum at 3 and 27 months of age in male and female mice. Data are presented as means ± SEM of 5–7 mice. Daggers (†) represent statistically significant difference from the value at 3 months of age during aging of male mice. Double daggers (‡) represent significant difference from the value at 3 months of age during aging of female mice. Age-dependent differences were considered at *p*<0.05 by one-way ANOVA, followed by Duncan's post-hoc test. Asterisks (*) represent statistically significant difference between male and female mice at respective ages during aging (*p*<0.05), by student t-test. In panel B, pound signs (#) represent differences of BA proportions between 3 and 27 months.

In male mice, the concentrations of most conjugated BAs (T-CA, T-αMCA, T-βMCA, T-UDCA, and T-HDCA) remained relatively constant during aging (Fig. 3A). In female mice, the concentrations of three BAs increased markedly between 3 and 27 months, namely T-βMCA (610%), T-UDCA (470%), and T-ωMCA (260%). However, the concentrations of T-CA, T-αMCA, T-DCA, T-MDCA, and T-HDCA remained relatively constant with age.

The composition of conjugated BAs in serum changed during aging (Fig. 3B). In male mice, the proportions of T-βMCA (11.8%→19.1%) and T-MDCA (3.1%→8.7%) increased, whereas T-DCA (12.4%→5.2%) and T-HDCA (2.4%→1.6%) decreased between 3 and 27 months of age. In female mice, the proportions of T-βMCA (13.0%→25.9%) and T-UDCA (1.8%→2.8%) increased, whereas T-MDCA (2.5%→1.2%) and T-DCA (8.1%→4.3%) decreased between 3 and 27 months.

### Unconjugated BAs in Serum during Aging

**Figure 4:**
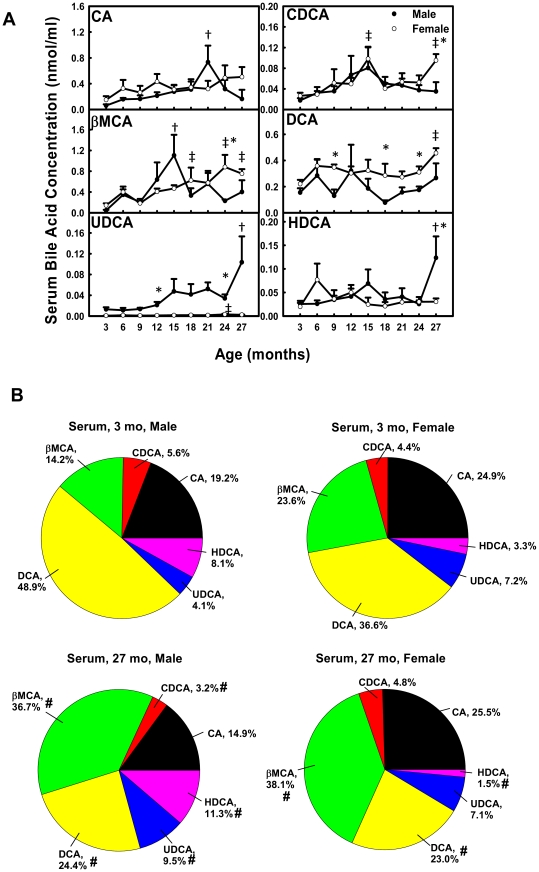
Concentrations of unconjugated BAs in serum during aging of male and female mice. (A) Concentrations of individual unconjugated BAs in serum during aging and (B) proportions of them in all unconjugated BAs in serum at 3 and 27 months of age in male and female mice. Data are presented as means ± SEM of 5–7 mice. Daggers (†) represent statistically significant difference from the value at 3 months of age during aging of male mice. Double daggers (‡) represent significant difference from the value at 3 months of age during aging of female mice. Age-dependent differences were considered at *p*<0.05 by one-way ANOVA, followed by Duncan's post-hoc test. Asterisks (*) represent statistically significant difference between male and female mice at respective ages during aging (*p*<0.05), by student t-test. In panel B, pound signs (#) represent differences of BA proportions between 3 and 27 months.

In male mice, the concentration of UDCA increased 800% from 3 to 27 months of age (Fig. 4A). CA increased (12.1-fold) from 3 to 21 months and decreased thereafter. Similarly, βMCA increased 24.6-fold from 3 to 15 months and decreased thereafter, and HDCA increased 480% from 3 to 27 months and decreased thereafter. CDCA and DCA remained relatively constant with age. In female mice, gradual age-dependent increase in concentrations of βMCA (530%) and DCA (210%) was observed between 3 and 27 months. CDCA was higher at 15 (370%) and 27 (360%) months than at 3 months of age. UDCA was 430% higher at 24 than 3 months. CA and HDCA remained relatively constant with age.

The composition of unconjugated BAs in serum also changed during aging (Fig. 4B). In male mice, the proportions of βMCA (14.2%→36.7%), UDCA (4.1%→9.5%), and HDCA (8.1%→?>11.3%) increased, whereas CDCA (5.6%→3.2%) and DCA (48.9%→24.4%) decreased between 3 and 27 months of age. In female mice, the proportion of βMCA (23.6%→38.1%) increased, whereas DCA (36.6%→23.0%) and HDCA (3.3%→1.5%) decreased between 3 and 27 months.

### Total BA Concentrations in Liver during Aging

**Figure 5:**
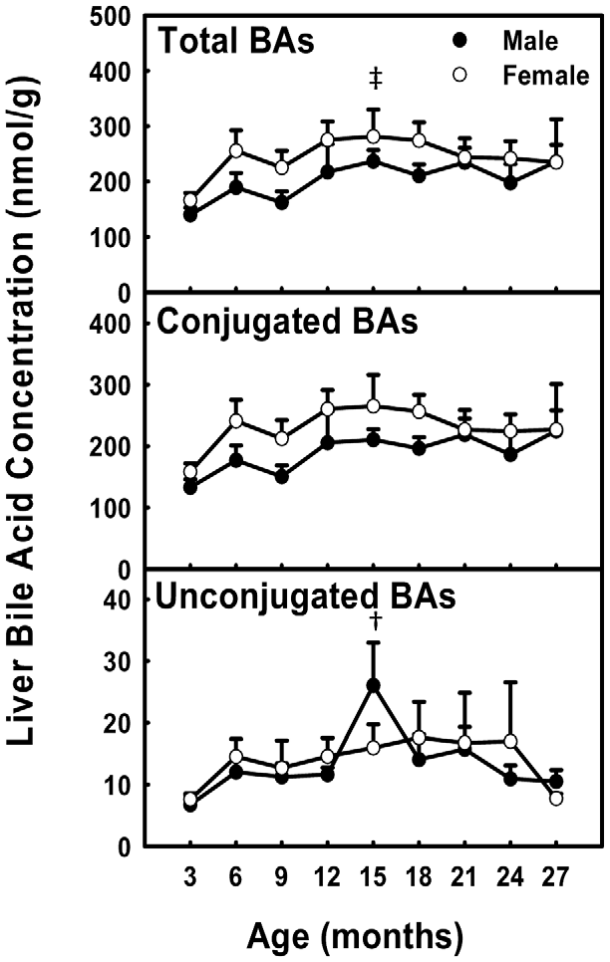
Total BA concentrations in liver during aging of male and female mice. Data are presented as means ± SEM of 5–7 mice. Daggers (†) represent statistically significant difference from the value at 3 months of age during aging of male mice. Double daggers (‡) represent significant difference from the value at 3 months of age during aging of female mice. Age-dependent differences were considered at *p*<0.05 by one-way ANOVA, followed by Duncan's post-hoc test. Asterisks (*) represent statistically significant difference between male and female mice at respective ages during aging (*p*<0.05), by student t-test.

The concentrations of total, conjugated, and unconjugated BAs in liver remained relatively constant with age, except a small increase (170%) of total BAs at 15 months of age in female mice, and a small increase (390%) of unconjugated BAs at 15 months in male mice (Fig. 5).

### Conjugated BAs in Liver during Aging

**Figure 6:**
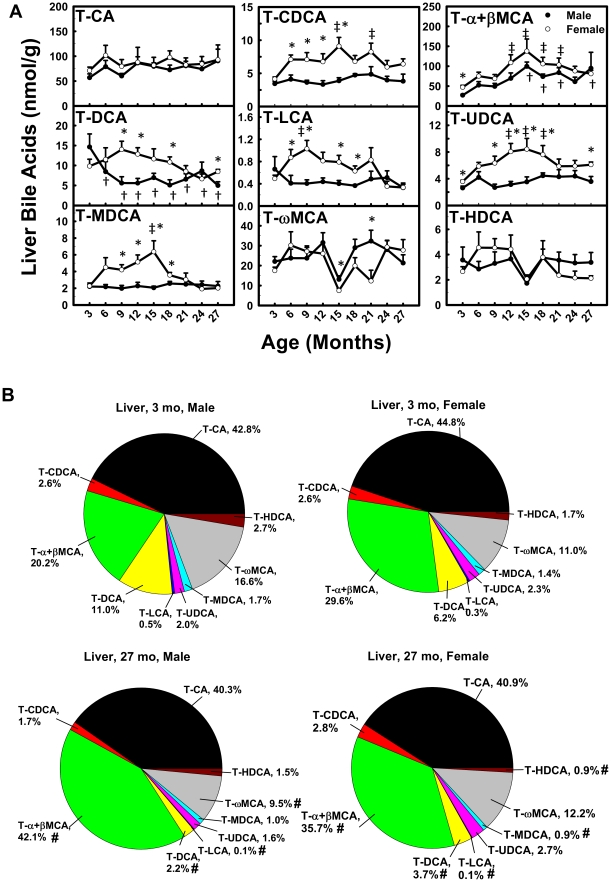
Concentrations of conjugated BAs in liver during aging of male and female mice. (A) Concentrations of individual conjugated BAs in liver during aging and (B) proportions of them in all conjugated BAs in liver at 3 and 27 months of age in male and female mice. Data are presented as means ± SEM of 5–7 mice. Daggers (†) represent statistically significant difference from the value at 3 months of age during aging of male mice. Double daggers (‡) represent significant difference from the value at 3 months of age during aging of female mice. Age-dependent differences were considered at *p*<0.05 by one-way ANOVA, followed by Duncan's post-hoc test. Asterisks (*) represent statistically significant difference between male and female mice at respective ages during aging (*p*<0.05), by student t-test. In panel B, pound signs (#) represent differences of BA proportions between 3 and 27 months.

In male mice, the concentration of T-α+βMCA gradually increased (350%), whereas T-DCA gradually decreased (65.8%) between 3 and 27 months of age (Fig. 6A). T-CA, T-CDCA, T-LCA, T-UDCA, T-MDCA, T-ωMCA, and T-HDCA remained relatively constant with age. In female mice, the concentrations of T-CDCA, T-α+βMCA, T-UDCA, and T-MDCA doubled between 3 and 15 months, and decreased thereafter. Similarly, T-LCA doubled between 3 and 9 months and decreased thereafter. T-CA, T-DCA, T-ωMCA, and T-HDCA remained relatively constant with age.

The composition of conjugated BAs in liver changed during aging (Fig. 6B). In male mice, the proportion of T-α+βMCA (20.2%→42.1%) increased, whereas T-DCA (11.0%→2.2%), T-LCA (0.5%→0.1%), and T-ωMCA (16.6%→9.5%) decreased between 3 and 27 months of age. In female mice, the proportion of T-α+βMCA (29.6%→35.7%) increased, whereas T-DCA (6.2%→3.7%), T-LCA (0.3%→0.1%), T-MDCA (1.4%→0.9%), and T-HDCA (1.7%→0.9%) decreased between 3 and 27 months.

### Unconjugated BAs in Liver during Aging

**Figure 7:**
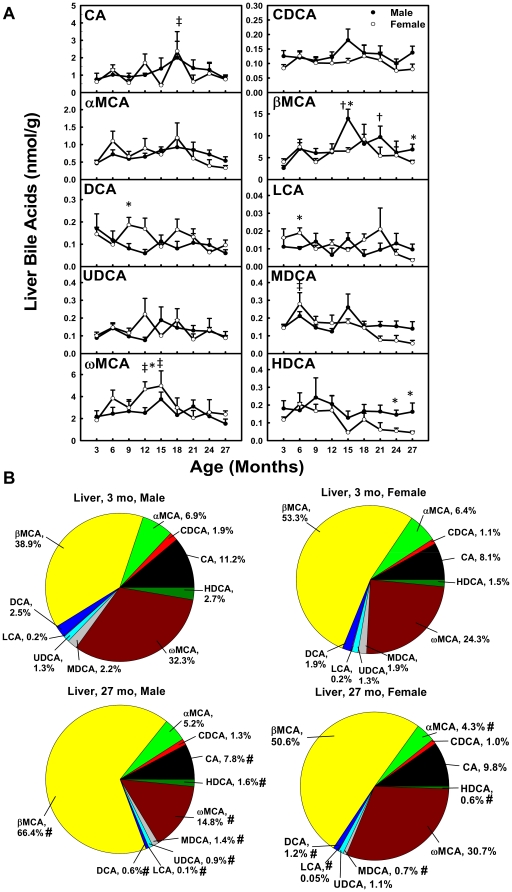
Concentrations of unconjugated BAs in liver during aging of male and female mice. (A) Concentrations of individual unconjugated BAs in liver during aging and (B) proportions of them in all unconjugated BAs in liver at 3 and 27 months of age in male and female mice. Data are presented as means ± SEM of 5–7 mice. Daggers (†) represent statistically significant difference from the value at 3 months of age during aging of male mice. Double daggers (‡) represent significant difference from the value at 3 months of age during aging of female mice. Age-dependent differences were considered at *p*<0.05 by one-way ANOVA, followed by Duncan's post-hoc test. Asterisks (*) represent statistically significant difference between male and female mice at respective ages during aging (*p*<0.05), by student t-test. In panel B, pound signs (#) represent differences of BA proportions between 3 and 27 months.

In male mice, The concentration of βMCA increased (530%) from 3 to 15 months and decreased 50.8% thereafter (Fig. 7A). In female mice, CA was 390% higher at 18 than 3 months, and ωMCA increased (170%) between 3 and 15 months and decreased 52.1% thereafter. CDCA, αMCA, DCA, LCA, UDCA, and HDCA remained relatively constant with age in both genders.

The composition of unconjugated BAs in liver changed during aging (Fig. 7B). In male mice, the proportion of βMCA (38.9%→66.4%) increased, whereas CA (11.2%→7.8%), DCA (2.5%→0.6%), LCA (0.2%→0.1%), UDCA (1.3%→0.9%), MDCA (2.2%→1.4%), ωMCA (32.3%→14.8%), and HDCA (2.7%→1.6%) decreased between 3 and 27 months. In female mice, αMCA (6.4%→4.3%), DCA (1.9%→1.2%), LCA (0.2%→0.05%), MDCA (1.9%→0.7%), and HDCA (1.5%→0.6%) decreased between 3 and 27 months.

### The mRNAs and Proteins of BA Hepatic Transporters during Aging

**Figure 8:**
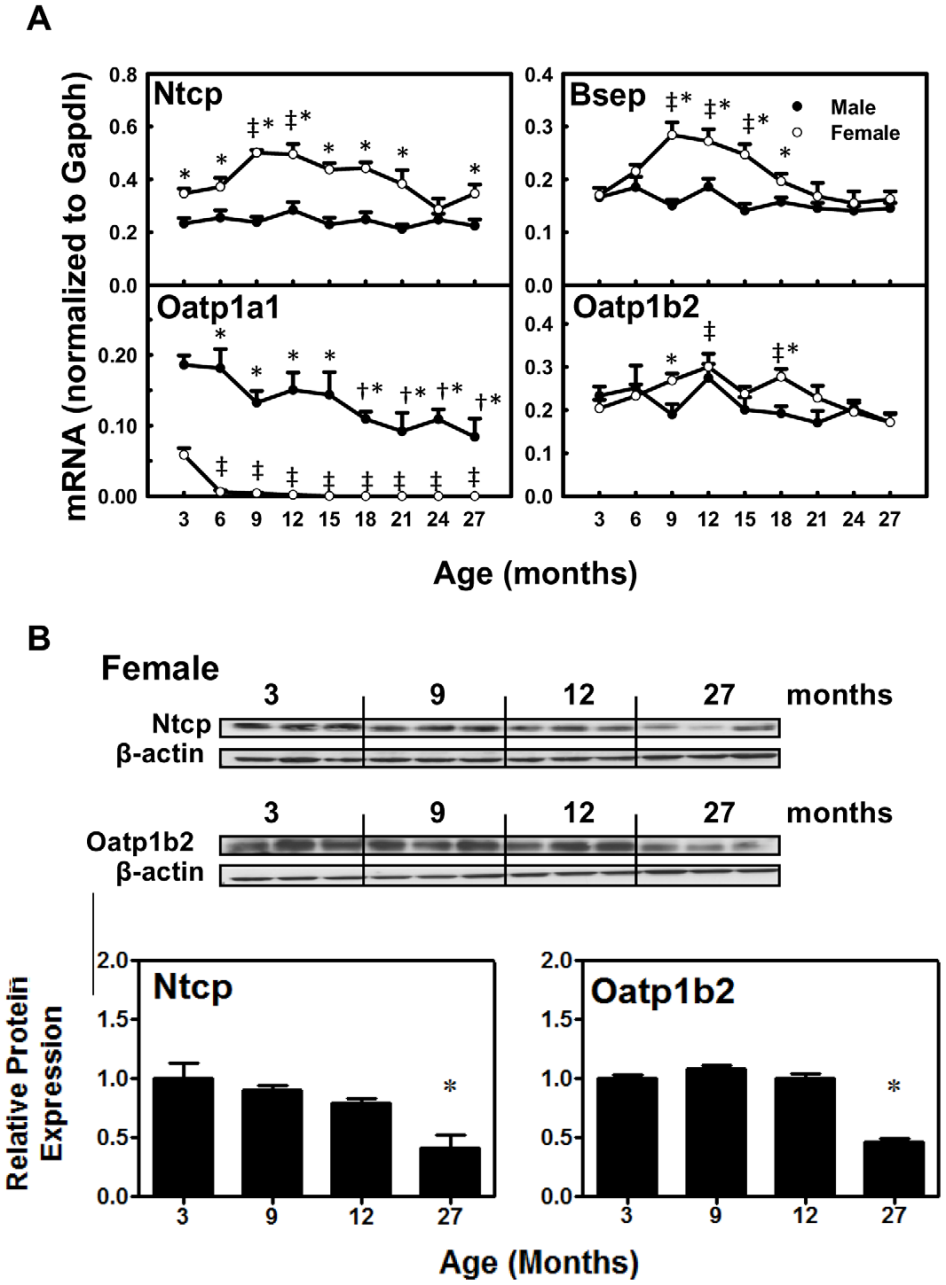
The mRNAs and proteins of BA transporters in livers during aging of male and female mice. A. The mRNAs of Ntcp, Bsep, Otap1a1, and Oatp1b2 during aging in male and female livers. Data are presented as means ± SEM of 5–7 mice, by normalization to Gapdh mRNA. B. Protein abundance of Ntcp and Oatp1b2 in several representative ages in female livers. Western blots for Ntcp (∼50 kDa) and Oatp1b2 (∼75 kDa) were performed using liver membrane protein fractions (40 µg protein/lane) from livers of female mice at 3, 9, 12, and 27 months of age. β-Actin (∼45 kDa) was used as loading control for each transporter. Bars represent the relative protein expression ± SEM of 3 mice. Daggers (†) represent statistically significant difference from the value at 3 months of age during aging of male mice. Double daggers (‡) represent significant difference from the value at 3 months of age during aging of female mice. Age-dependent differences were considered at *p*<0.05 by one-way ANOVA, followed by Duncan's post-hoc test. Asterisks (*) represent statistically significant difference between male and female mice at respective ages during aging (*p*<0.05), by student t-test.

The mRNAs of BA uptake transporter Ntcp and efflux transporter Bsep remained relatively constant with age (Fig. 8A). Whereas they shared an age-dependent expression pattern in female mice, which was first an increase from 3 to 9 months (Ntcp: 140%; Bsep: 170%) and then a decrease from 9 to 27 months (Ntcp: 30.8%; Bsep: 42.8%). The mRNA of uptake transporter Oatp1b2 for unconjugated BAs remained constant with age in male mice, whereas in female mice, it increased 150% from 3 to 12 months and decreased 43% thereafter. The mRNA of male-predominantly expressed uptake transporter Oatp1a1 in male mice decreased gradually (54.9%) between 3 and 27 months. Interestingly, Oatp1a1 mRNA in female mice decreased markedly (89.4%) from 3 to 6 months and remained extremely low thereafter.

As shown in Fig. 8B, decreased proteins in female livers were observed by western blots at 27 months for Ntcp (59%) and Oatp1b2 (53.4%), confirming the decreased expression of these two uptake transporters at a later stage of life in female mice.

### The mRNAs of BA Synthetic Enzymes in Liver during Aging

**Figure 9:**
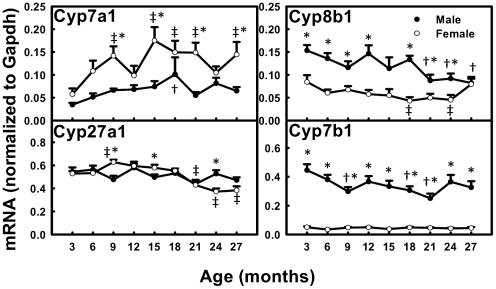
The mRNAs of BA synthetic enzymes in livers during aging of male and female mice. Data are presented as means ± SEM of 5–7 mice, by normalization to Gapdh mRNA. Cyp, cytochrome P450. Daggers (†) represent statistically significant difference from the value at 3 months of age during aging of male mice. Double daggers (‡) represent significant difference from the value at 3 months of age during aging of female mice. Age-dependent differences were considered at *p*<0.05 by one-way ANOVA, followed by Duncan's post-hoc test. Asterisks (*) represent statistically significant difference between male and female mice at respective ages during aging (*p*<0.05), by student t-test.

The mRNA of the rate-limiting enzyme Cyp7a1 for BA synthesis increased 290% from 3 to 18 months in male mice and tended to decrease thereafter, whereas in female mice, it increased 300% from 3 to 15 months and remained high thereafter (Fig. 9). Cyp8b1 decreased 46.5% between 3 and 27 months in male mice, whereas in female mice, it decreased 46.6% between 3 and 24 months, and then increased thereafter. Cyp27a1 remained relatively constant with age in male mice, whereas in female mice, it increased 120% from 3 to 9 months and then decreased 39.0% thereafter. Cyp7b1 decreased 43.8% between 3 and 21 months and tended to increase thereafter in male mice, whereas it was relatively constant with age in female mice.

### The mRNAs of Regulators of Cyp7a1 Transcription during Aging

**Figure 10:**
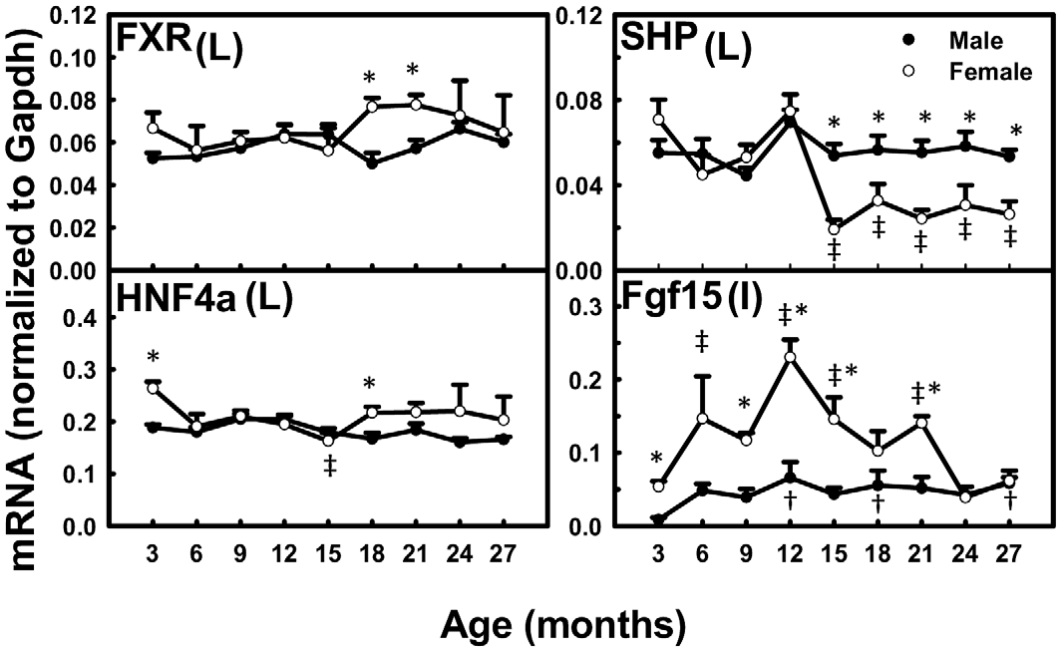
The mRNAs of regulators of Cyp7a1 transcription during aging of male and female mice. Figure shows mRNA data for FXR, SHP, and HNF4α in liver (L) and Fgf15 in ileum (I). Data are presented as means ± SEM of 5–7 mice, by normalization to Gapdh mRNA. Daggers (†) represent statistically significant difference from the value at 3 months of age during aging of male mice. Double daggers (‡) represent significant difference from the value at 3 months of age during aging of female mice. Age-dependent differences were considered at *p*<0.05 by one-way ANOVA, followed by Duncan's post-hoc test. Asterisks (*) represent statistically significant difference between male and female mice at respective ages during aging (*p*<0.05), by student t-test.

In liver, the mRNA of FXR and HNF4α remained relatively constant with age in both genders (Fig. 10). SHP was relatively constant with age in male mice, whereas in female mice, it markedly decreased (74.1%) from 12 to 15 months and remained low thereafter. In ileum, the mRNA of Fgf15 gradually increased 670% between 3 and 27 months in male mice, whereas in female mice, it increased 430% between 3 and 12 months and decreased 73.3% thereafter.

## Discussion

The present study demonstrates a female-specific increase of total BA concentrations in serum during aging of mice. Both conjugated and unconjugated BAs in serum increase with age in female mice, whereas they remain relatively constant with age in male mice (Fig. 2). It has been reported that there were no age-related changes between young (8 weeks) and middle-aged (12 months) male rats in biliary BA secretion, distribution of BAs in the bile and intestine, nor the turnover frequency of BAs [22], and in addition, the biliary BA secretion in aged (106 weeks) male rats was similar to younger rats [23]. The finding of the present study that concentrations of total BAs in both serum and liver are similar in young and aged male mice is consistent with the previous reports in aged male rats. In humans, fasting concentrations of conjugated and unconjugated serum BAs were similar between 12 younger (mean age 37 years, range 22–59 years; seven female, five male) and 12 elder (mean age 67 years, range 60–82 years; seven female, five male) subjects [24]. This finding in humans is different from the present results in mice, possibly because 1) there are species differences in BA composition and BA conjugation between humans and mice, 2) the small number of subjects compromises the conclusion from the human study, and 3) possible changes of BAs in female subjects may be missed because male and female subjects were not analyzed separately in the human study.

Total BAs in serum and liver become more hydrophilic during aging in both male and female mice. Heuman [25] and Wang et al. [26] have reported the order of hydrophobicity indices of individual BAs as follows: T-ωMCA (∼−0.9)<T-αMCA (−0.84)<T-βMCA (−0.78)<T-MDCA (∼−0.6)<T-UDCA (−0.47)<T-HDCA (−0.35)<T-CA (0.00)<T-CDCA (+0.46)<T-DCA (+0.59)<T-LCA (+1.00), ωMCA (∼−0.77)<αMCA (∼−0.7)<βMCA (∼−0.65)<MDCA (∼−0.47)<UDCA (−0.31)<HDCA (∼−0.22)<CA (+0.13)<CDCA (+0.59)<DCA (+0.72)<LCA (+1.13). The hydrophobicity index (HI) of individual BAs and their proportions in biological samples are used to predict the HI of total BAs at physiological *p*H. Total BAs become more hydrophilic during aging, because the HI of total BAs decreases between 3 and 27 months in serum (M: −0.096→−0.24; F: −0.095→−0.20) and liver (M: −0.27→−0.42; F: −0.32→−0.38). The decrease is largely due to the increased proportion of hydrophilic βMCA and decreased proportion of hydrophobic DCA (Figs. 3B, 4B, 6B, and 7B).

The changes of individual BAs during aging in the present study provide important evidence that BAs may function as markers for longevity. A very intriguing finding in the long-lived lit/lit mice is that they had increased concentrations of several BAs in the serum, and feeding CA to wild-type mice reproduced the expression profiles of xenobiotic metabolism genes observed in the long-lived mice, which might increase resistance to stress and alleviate age-related tissue damage [27]. Females had a longer life expectancy than males in many species, including humans [28]. As to laboratory animals, female Wistar rats lived on average 14% longer than males, and female BALB/cJ mice lived much longer than males. The median life span of female mice in a heterogeneous background was shown to be longer than males at three research sites [29]. C57BL/6J mice are long-lived mice compared to other inbred strains, and females tended to live longer than males, but this was not statistically significant, possibly due to the small number of animals [30]. In the present study, serum concentrations of T-CA, T-αMCA, T-βMCA, T-DCA, and T-UDCA are higher in female C57BL/6 mice, and increase during aging in female mice (Fig. 3A). The long-lived lit/lit mice were shown to have increased βMCA, CDCA, LCA, DCA, CA, and UDCA in serum [27]. Therefore, increased serum concentrations of some individual BAs, such as CA, βMCA, DCA, and UDCA might correlate with the tendency of increased longevity in female C57BL/6 mice. UDCA is used clinically to treat gallstones. In cancer cells, UDCA induced senescence through increased histone hypoacetylation and inhibiting telomerase activity [31]. In the present study, T-UDCA in serum increases 470% between 3 and 27 months of age in female mice (Fig. 3A). UDCA in serum increases 800% between 3 and 27 months in male mice, and increases 430% between 3 and 24 months in female mice (Fig. 4A). The increased serum concentrations of UDCA during aging indicate that UDCA in serum may be an important marker of longevity.

Interesting findings from the present study show gender-divergent changes of expression of BA hepatic uptake and efflux transporters during aging of mice. In female mice, BA uptake transporters Ntcp and Oatp1b2 have decreased expression from about 12 to 27 months (Fig. 8A and 8B), and the BA efflux transporter Bsep has decreased expression from 9 to 27 months of age (Fig. 8A). In contrast, in male mice, the expression of Ntcp, Oatp1b2, and Bsep remains constant during aging. Female-predominant Ntcp expression in young adult mice is due to the inhibitory effect of male-pattern growth hormone secretion [32]. The lower sodium-dependent uptake of taurocholate in hepatocytes from female rats was partially due to female-specific lower expression of Ntcp [33]. The gender-divergent expression changes of Ntcp and Oatp1b2 during aging in the present study might be regulated by gender differences in growth hormone secretion patterns, or changes of female sex hormones with the cease of rodent estrous cycles. In contrast with the changes of BA uptake transporters, the mRNAs of BA-conjugating enzymes (BAL and BAT) and ileal BA transporters (Asbt and Ostα/β) remain relatively constant with age in both genders (data not shown). Decreased expression of uptake transporters Ntcp and Oatp1b2 likely contributes to the accumulation of BAs in serum with aging in female mice.

In addition to the decreased expression of BA uptake transporters, increased Cyp7a1 expression probably contributes to the increased concentrations of total BAs in serum during aging in female mice. The present study shows Cyp7a1 mRNA increases from 3 to 9 months of age, and remains at a high level thereafter in female mice, whereas in male mice, it remains constant during aging (Fig. 9). However, the present data are opposite to previous reports in human livers showing decreased cholesterol 7α-hydroxylation in the elderly [34], [35] and an inverse correlation between age and CYP7A1 mRNA levels [36] in both male and female subjects. There are probably species differences in BA changes during aging between human and mice, and the reliability of the findings in the human study is compromised by the low correlation coefficient (about −0.6) and the small number of subjects.

The present study characterizes the mRNA changes of several regulators for Cyp7a1 transcription during aging. SHP mRNA remains at low levels from 15 to 27 months compared to that at 3 months in female mice, and is inversely correlated with Cyp7a1 mRNA during aging in female mice (Fig. 10). This can be explained by the down-regulation of Cyp7a1 transcription by the FXR-SHP pathway. The Fgf15 signaling is also important for down-regulating Cyp7a1 transcription. Mice with liver-conditional knockout of HNF4α have reduced mRNA of Cyp7a1 [37], indicating that HNF4α is probably a positive regulator for Cyp7a1 transcription in mice. However, the mRNAs of neither Fgf15 nor HNF4α correlate with the elevated Cyp7a1 mRNA (Fig. 10). Therefore, in the present models, we conclude that Cyp7a1 transcription appears to be regulated by SHP inhibition during aging.

The current study provides a comprehensive description of the age-related changes of BA composition and concentration in serum and liver of male and female C57BL/6 mice from 3 to 27 months of age. The major findings are that (1) total BA concentrations in serum increase during aging in female mice, whereas they remain relatively constant in male mice; (2) livers maintain constant concentrations of BAs during aging in both genders; (3) total BAs in serum and liver become more hydrophilic during aging in both genders, largely due to the increased proportion of βMCA and decreased proportion of DCA between 3 and 27 months; (4) mRNAs of Ntcp and Oatp1b2 decrease from 9 to 27 months in female mice, whereas they remain relatively constant in male mice; (5) Cyp7a1 mRNA increases from 3 to 9 months and remains at high levels thereafter in female mice, which inversely correlates with SHP mRNA during aging. In male mice, however, Cyp7a1 mRNA remains constant with age. Therefore, the female-specific increased total BAs in serum during aging appear to result from a female-specific BA-related gene expression pattern, which is the decreased BA uptake transporters, Ntcp and Oatp1b2, and the increased rate-limiting enzyme for BA synthesis Cyp7a1.
